# Rhenium Alkyne Catalysis: Sterics Control the Reactivity

**DOI:** 10.1021/acs.inorgchem.3c04235

**Published:** 2024-03-20

**Authors:** Michele Tomasini, Martí Gimferrer, Lucia Caporaso, Albert Poater

**Affiliations:** † Institut de Química Computacional i Catàlisi, Departament de Química, Universitat de Girona, c/Ma Aurèlia Capmany 69, Girona 17003, Catalonia, Spain; ‡ Dipartimento di Chimica e Biologia, Università di Salerno, Via Ponte don Melillo, Fisciano 84084, Italy; § Institut für Physikalische Chemie, Georg-August Universität Göttingen, Tammannstraße 6, Göttingen 37077, Germany; §§ CIRCC, Interuniversity Consortium Chemical Reactivity and Catalysis, via Celso Ulpiani 27, Bari 70126, Italy

## Abstract

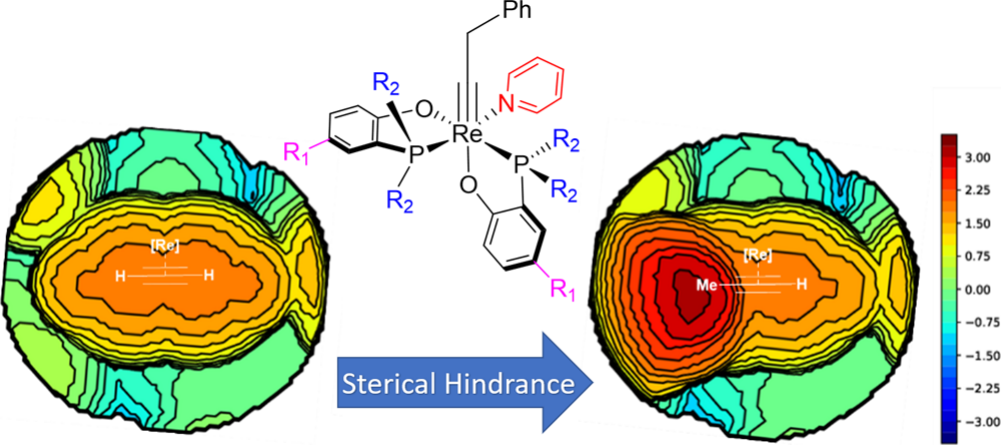

Metathesis reactions, including alkane, alkene, and alkyne metatheses, have their origins in the fundamental understanding of chemical reactions and the development of specialized catalysts. These reactions stand as transformative pillars in organic chemistry, providing efficient rearrangement of carbon–carbon bonds and enabling synthetic access to diverse and complex compounds. Their impact spans industries such as petrochemicals, pharmaceuticals, and materials science. In this work, we present a detailed mechanistic study of the Re(V) catalyzed alkyne metathesis through density functional theory calculations. Our findings are in agreement with the experimental evidence from Jia and co-workers and unveil critical factors governing catalyst performance. Our work not only enhances our understanding of alkyne metathesis but also contributes to the broader landscape of catalytic processes, facilitating the design of more efficient and selective transformations in organic synthesis.

## Abstract 2

The sterical hindrance of alkyne substrates control the Re catalysts.

## Introduction

Metathesis reactions are pivotal transformations involving the reshuffling of carbon–carbon (C–C) bonds of different multiplicities—namely, single, double, and triple C–C bonds for alkane, alkene, and alkyne metathesis, respectively. Alkane metathesis, for instance, entails the redistribution of single C–C bonds within alkanes, yielding a different alkane product. This transformation traces its roots to pioneering work in novel catalyst design capable of selectively breaking and reassembling alkane C–C bonds, paving the way for subsequent advancements.^1,2^ These seminal studies not only unveiled the potential of alkane metathesis in converting simple hydrocarbons into more intricate ones but also underscored its applicability across diverse sectors, e.g., the petrochemical industry.

**Scheme 1:**
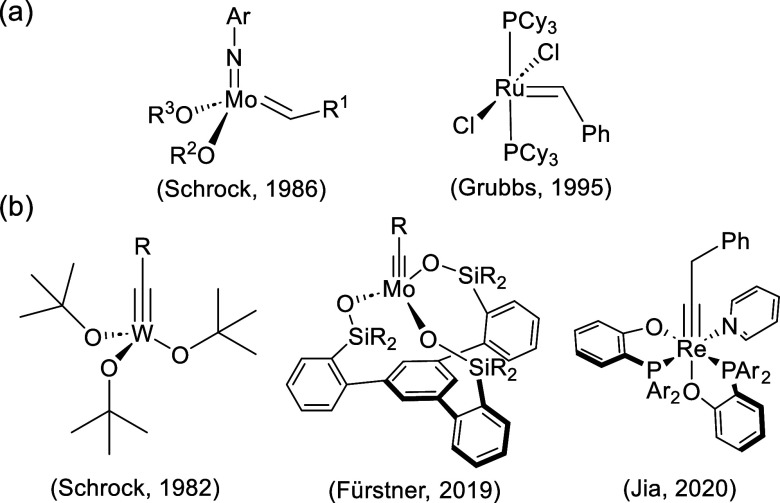
Reference Catalysts for Alkene Metathesis (a), and Example Catalysts for Alkyne Metathesis (b)

Similarly, alkene metathesis involves the reorganization of C–C double bonds in alkenes, originating in the early 1970s with Chauvin’s elucidation of its mechanistic intricacies.^3^ Subsequent breakthroughs by Grubbs and Schrock yielded efficient catalysts for this reaction, facilitating its practical application (Scheme 1a).^4,5^ Alkyne metathesis, albeit to a lesser extent,^6,7^ emerged as a versatile strategy for synthesizing complex molecules, finding utility in various organic chemistry procedures,^8,9^ including ring-opening alkyne polymerization,^10^ and the synthesis of conjugated polymers via metathesis of acyclic diynes.^11,12^

In the 1980s, Schrock and co-workers developed the first homogeneous catalysts for alkyne metathesis (Scheme 1b),^13^ based on well-defined d^0^ W(VI) and Mo(VI) alkylidyne catalysts. This marked a departure from earlier heterogeneous catalysts consisting of WO_3_/silica, which operated only under high temperatures exceeding 200 °C.^14^ Subsequent homogeneous catalysts were reported, involving a mixture of Mo(CO)_6_ and phenols, capable of functioning in high-boiling-point solvents (around 150 °C).^15^ Due to their activity and stability, Mo-based catalysts garnered increasing interest, resulting in the development of highly efficient d^0^ W(VI)/Mo(VI) alkylidyne catalysts with customizable ligand systems, encompassing fluorinated alkoxides,^16^ silanolates,^17^ and unconventional combinations of electron-withdrawing alkoxides with imido,^18^ amido,^19^ silanolate,^20^ and NHC ligands.^21^

Chronologically, Mo(VI) alkylidyne precursors with monodentate^22^ or polydentate^23^ alcohol preceded the development of Mo(VI)/W(VI) canopy catalysts with a tridentate silanolate ligand, pioneered by the Fürstner^24^ and Lee research groups.^25^ These catalysts exhibited remarkable compatibility with protic functional groups, even in the presence of traces of water. Subsequently, the Zhang group reported in situ-formed catalytic systems capable of performing alkyne metathesis under open-air conditions.^26^

**Scheme 2:**
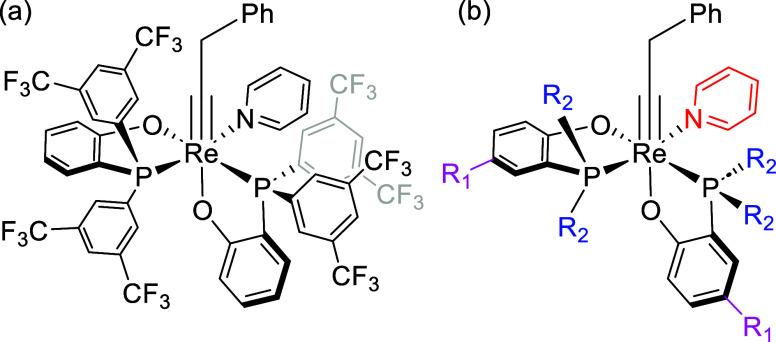
d^2^ Re(V) Alkylidyne Complexes for Catalytic Alkyne Metathesis Synthesized in (a) 2020 and (b) 2022 by Williams, Jia, and Co-Workers^30,31^

Beyond the traditional d^0^ Mo/W systems, Schrock^27^ proposed non-d^0^ transition-metal (TM) alkylidyne complexes,^28^ enhancing the catalysts stability and substrate scope. Note that examples of this type of systems increased substantially in the last years.^29^ Since the insights reported by Ehrhorn and Tamm,^7^ this challenge remained unexplored until 2020 with the disclosure of a d^2^ Re(V) alkylidyne by Williams, Jia, and co-workers. This complex exhibited low activity yet remarkable air stability, catalyzing alkyne metathesis in the presence of traces of water and demonstrating compatibility with a wide range of functional groups (see Scheme 2a).^30^

Subsequent work by the same team unveiled a series of Re(V) alkylidyne complexes featuring phosphino-phenolate (PO) bidentate ligands,^31^ each possessing distinct electronic and steric properties.^32^ Significantly, this research achieved a noteworthy milestone by showcasing, for the first time, the catalysis of non-d^0^ alkylidyne complexes in ring-closing alkyne metathesis (RCAM) for the first time. This breakthrough allows establishing a clear dependence between the high activity of these catalysts and their structural characteristics within this family of systems. This correlation sheds light on the factors influencing their performance in alkyne metathesis reactions. However, a complete understanding of the mechanism remains elusive, prompting the need for detailed investigations.

Motivate by these advancements, in this work, we unveil the mechanism of alkyne metathesis catalyzed by a formally d^2^ Re(V) alkylidyne complex (see Scheme 2b, with R_1_ = H and R_2_ = Ph) by means of density functional theory (DFT) calculations. Moreover, we investigate the steric and electronic properties of differently substituted PO ligands and their impact on the catalytic activity, aiming to enhance predictive capabilities in Re-based alkyne catalysts design.^33^

## Computational Details

All DFT calculations were performed with the Gaussian16 package.^34^ Geometry optimizations were performed using the BP86 functional, a pure GGA functional developed by Becke and Perdew,^35^ including the Grimme D3 dispersion correction. These calculations were performed in conjunction with the all electron double-ζ polarized def2-SVP basis set for light atoms,^36^ whereas for Re the SDD basis set (and pseudopotential) has been employed.^37^ All optimizations were carried out without symmetry constraints, and the nature of the stationary points was confirmed by analytical frequency analysis. Gibbs free energies at 373.15 K were calculated using the electronic energy evaluated with the M06 functional,^38^ and the triple-ζ basis set def2-TZVP for all the atoms,^39^ except for Re that used again the SDD basis set (and pseudopotential). Furthermore, solvent effects were estimated with the universal solvation model SMD from Cramer and Truhlar using toluene as solvent.^40^ The reported Gibbs free energies encompass electronic energies obtained at the M06/def2-TZVP(SMD(toluene))∼SDD//BP86-D3/def2-SVP∼SDD level of theory. These values were corrected with zero-point vibrational energies, thermal corrections, and entropy effects computed at the BP86-D3/def2-SVP∼SDD level.

## Results and Discussion

**Figure 1:**
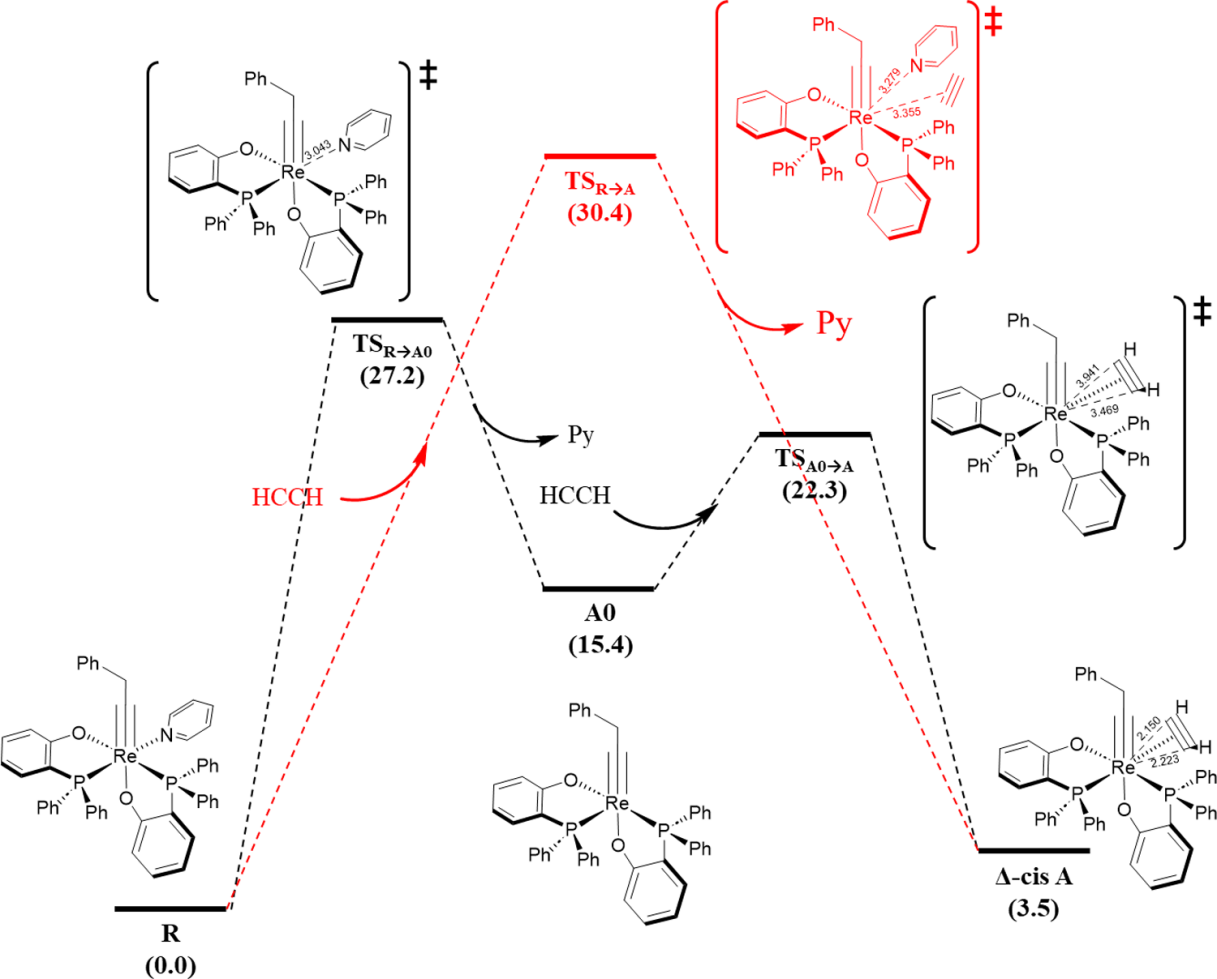
Reaction pathway for exchange of the pyridine (py) by the alkyne substrate on the rhenium complex **R** (relative Gibbs free energies in kcal/mol and selected distances in Å).

**Figure 2:**
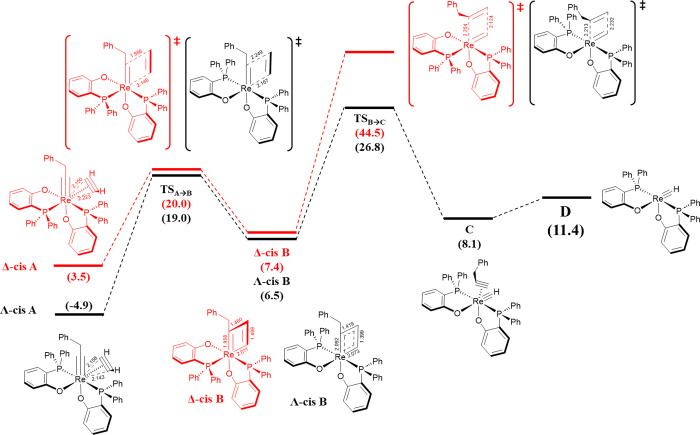
Activation pathway for the formation of the rhenium complex **D**, the catalytic active species for alkyne metathesis (relative Gibbs free energies in kcal/mol and selected distances in Å).

Let us start with the mechanistic study of the replacement of a pyridine ligand from precatalyst species **R** by an acetylene molecule. For this rather simple process, two possible pathways exist, associative and dissociative, being both considered and evaluated. The results for both pathways, including the relative Gibbs energies of the involved species obtained at the M06/def2-TZVP∼SDD(SMD(toluene))//BP86-D3/def2-SVP∼SDD level of theory, are gathered in Figure 1. In the associative pathway, the acetylene molecule first approaches the precatalyst species **R**, provoking the Re–N bond breaking and coordinating in a η^2^-fashion, forming species **A** (Δ*G* = 3.5 kcal/mol) through a hepta-coordinated transition state **TS**_**R→A**_ (Δ*G*^‡^ = 30.4 kcal/mol). This strained transition state suffers the π-conflict paradox, i.e., the repulsion between pπ electrons of the entering alkyne and the M–C triple bond dπ electrons.^41^ Alternatively, the dissociation pathway (Figure 1) involves the formation of species **A** in two steps. First, the pyridine ligand is released (to the solvent media) via **TS**_**R→A0**_ (Δ*G*^‡^ = 27.2 kcal/mol), forming the coordinatively unsaturated (vacant site present), unstable and thus highly reactive intermediate **A0** (Δ*G* = 15.4 kcal/mol). Then, the latter intermediate reacts with acetylene to form **A** through **TS**_**A0→A**_ (Δ*G*^‡^ = 22.3 kcal/mol). Overall, the associative mechanism, which is coincident with the concerted mechanism as well, through **TS**_**R→A**_ is kinetically unfavored (30.4 vs 27.2 kcal/mol) and not plausible under the reported reaction conditions (*T* = 100 °C), being ruled out. Instead, the octahedral alkyne–alkylidyne complex **A** is formed thanks to the pyridine ligand dissociation and the alkyne can work as a 4e-donor.^42^ Once **A** is formed, the reaction, i.e., the activation, can proceed (Figure 2) through a cycloaddition step leading to the rhenacyclobutadiene **B** (Δ*G* = 7.4 kcal/mol) via **TS**_**A→B**_ (Δ*G*^‡^ = 20.0 kcal/mol). However, the formation of species **C** from **B** requires an enormous amount of energy (Δ*G*^‡^_TSB→C_ = 44.5 kcal/mol), which is kinetically impossible. **A** is the Δ-*cis* isomer depicted in red in Figure 2, and its isomer **Λ-*cis*A** exists, depicted in black in Figure 2. Thermodynamically, the latter is 8.5 kcal/mol more stable than **Δ-*cis*A**, thus placed 4.9 kcal/mol below the reference complex **R**. More importantly, it can efficiently catalyze the reaction (Figure 2), since ΔΔ*G*^‡^_TSB→C_ = 17.7 kcal/mol, clearly favoring the **Λ** based one. Structurally the difference is that the two phosphorus atoms are placed *cis* in **Δ-*cis*A** and trans in **Λ-*cis*A**. Ignoring the axis with the coordinated alkyne, the average of the two remaining P–Re–P and C–Re–O axes for **Λ-*cis*A** is 166.0°, 2.0 closer to linearity than for **Δ-*cis*A**, across its P–Re–O and C–Re–O axes. In addition, although it may seem contradictory, the axis P–Re–P where the alkyne in **Λ-*cis*A** is inserted is 157.3° while it is 161.1° in the P–Re–O for **Δ-*cis*A**, that is, it being more difficult in the latter case to accommodate the alkyne, especially with sterically hindered substituents. In fact, the coordination of the alkyne is more symmetrical maintaining better the κ^2^ coordination for **Λ-*cis*A** with Re–C bond distances of 2.143 and 2.158 Å than for **Δ-*cis*A** (2.223 and 2.150 Å). The difference in stability of both isomers can also be rationalized by analyzing the frontier molecular orbitals (see Figures S1 and S2 in the SI). In **Λ-*cis*A**, no alkyne atomic orbital contribution is present to form the HOMO and LUMO while an orbital node is present in **Δ-*cis*A** LUMO in the region between Re and an alkyne carbon atom. As a consequence, one alkyne carbon is more negatively charged than the other in **Δ-*cis*A**, as shown by the NAO-obtained atomic charges (*q*_C1_ = −0.215 vs *q*_C2_ = −0.174). In contrast, the difference in atomic charge in **Λ-*cis*A** (*q*_C1_ = −0.225 vs *q*_C2_ = −0.228) is not as large, making the complex more stable. Furthermore, apart from the series of structural factors (distortion in coordination of acetylene, steric repulsion between phosphine groups, etc.) that contribute to the relative stability favoring **Λ-*cis*A**, the HOMO–LUMO gap is lower in **Λ-*cis*A** than in **Δ-*cis*A** (102.0 kcal/mol for **Λ-*cis*A** vs 103.7 kcal/mol for **Δ-*cis*A**). The nonplanarity of intermediate **B** can be rationalized by the HOMO and LUMO shapes. By visual inspection of the HOMO of **Δ-*cis*A**, one observes that the orbital lobes around the Re alkylidyne bond form an angle of 135° with the Re–P bond if visualized along the Re alkylidyne bond (top view in the SI). Hence, when the alkyne rotates to form rhenacyclobutadiene species **B**, the best overlap between the HOMO lobe centered at *C*_alkylidyne_ and the LUMO lobe centered at C1 will result in a nonplanar rhenacyclobutadiene. Then, the nonplanarity of **B** influences how the metal interacts with the neighboring atoms. For its analysis, we used the Mayer bond orders (MBOs).^43^ While the MBO of the Re–C_alkylidyne_ bond (MBO = 1.019 in **Δ-*cis*B** vs MBO = 1.787 in **Δ-*cis*A**) and MBO C1–C2 (MBO = 1.348 in **Δ-*cis*B** vs MBO = 2.176 in **Δ-*cis*A**) are lower than those corresponding ones to **Λ-*cis*A**,^44^ the C1–C_alkylidyne_ bond is far from being the ideal double bond (MBO = 1.130 in **Δ-*cis*B**).^45^ In contrast, starting from **Λ-*cis*A**, the reaction can proceed without the need to overcome a high activation energy barrier. Indeed, although intermediate **Λ-*cis*B** is formed after overcoming a higher activation energy barrier (23.9 kcal/mol, thus 3.9 kcal/mol more than the previous case) mainly due to the higher stabilization of **Λ-*cis*A**, the formation of intermediate **C** (Δ*G* = 8.1 kcal/mol) becomes kinetically feasible through **TS**_**B→C**_ (Δ*G*^‡^ = 26.8 kcal/mol). The lower required energy is due to the difference in MBO in **Λ-*cis*B** with respect to **Δ-*cis*B**. Particularly, the planarity of the rhenacyclobutadiene leads to a stronger C1–C_alkylidyne_ bond (MBO = 1.356 in **Λ-*cis*B** vs MBO = 1.787 in **Δ-*cis*B**) and weaker Re–C_alkylidyne_ (MBO = 0.701 in **Λ-*cis*B** vs MBO = 1.019 in **Δ-*cis*B**) and C1–C2 (MBO = 1.314 in **Λ-*cis*B** vs MBO = 1.348 in **Δ-*cis*B**).

**Figure 3:**
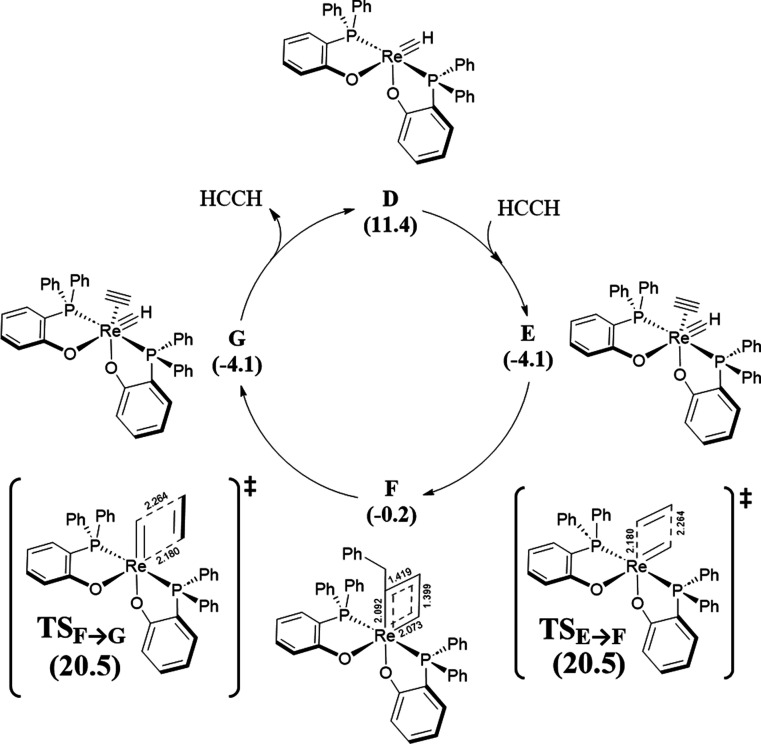
Re-based catalyzed pathway for alkyne metathesis (relative Gibbs free energies in kcal/mol with respect to **R**, and selected distances in Å).

**Figure 4:**
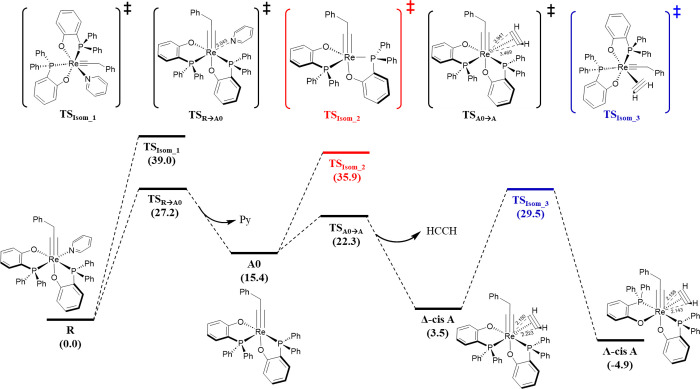
Re-based catalyzed pathway of the isomerization (relative Gibbs free energies are given in kcal/mol with respect to **R**).

From **C** a molecule of benzyl acetylene is released into the solution forming intermediate **D** (Δ*G* = 11.4 kcal/mol). The newly formed **D** bears a vacant site, and it can easily be coordinated by acetylene to form the η^2^-acetylene complex **E** (Δ*G* = −4.1 kcal/mol). Above with **A**, the reaction proceeds with a cycloaddition step to form the rhenacyclobutadiene **F** (Δ*G* = −0.2 kcal/mol) followed by a cycloelimination step (Figure 4). However, unlike what happens with **A**, **TS**_**E→F**_, and **TS**_**F→G**_ are isoenergetic (Δ*G*^‡^ = 20.5 kcal/mol) while **E** is symmetrical. Finally, intermediate **G** (Δ*G* = −4.1 kcal/mol) is formed and releases an acetylene molecule into the solution, forming again the unstable intermediate **D** (Δ*G* = 11.4 kcal/mol) ready to react with another acetylene molecule and continuing with the catalytic cycle (Figure 3). Once it was established that **Λ-*cis*A** kinetically favors the reaction, the isomerization mechanism from the Δ-*cis* isomer to a Λ-*cis* one has been studied (Figure 4). Isomerization can occur once the pyridine has left, forming **A0** or via an intramolecular twisted transition state apart from **R** or **A** without breaking any bond. Indeed, **R** can isomerize via a Rây–Dutt twisted **TS**_**Isom_1**_ (Δ*G*^‡^ = 39.0 kcal/mol).^46^ On the other hand, isomerization is more favored through a trigonal bipyramidal **TS**_**Isom_2**_ (Δ*G*^‡^ = 35.9 kcal/mol). In fact, the dissociation of the pyridine allows the rotation along the C–Re–O axis of one PO ligand, since the dissociation of pyridine frees up enough space to make that rotation feasible. To a lesser extent, but not least, the direct isomerization of **Δ-*cis*A** to **Λ-*cis*A** was analyzed. This isomerization mechanism is kinetically much more favored than the previous ones that occur via a Rây–Dutt twisted **TS**_**Isom_3**_ (Δ*G*^‡^ = 29.5 kcal/mol).

By applying the Kozuch–Shaik energetic span model theory to our catalytic cycle,^47^ an overall energetic span δ*E* of 34.4 kcal/mol is obtained, with **TS**_**Isom_3**_ being the TOF-determining transition state (TDTS) and **Λ-*cis*A** the TOF-determining intermediate (TDI). Once the TDTS and the TDI species were identified, a series of catalysts (Table 1, entries 1–7) were analyzed tuning the steric and electronic properties of the PO ligands. As shown in Table 1, the presence of a more electron-withdrawing phosphine (entry 2) does not influence the reactivity as much because the resulting stabilization of **TS**_**Isom_3**_ (ΔΔ*G*^‡^ = −1.7 kcal/mol) is compensated by the stabilization of **Λ-*cis*A** (ΔΔ*G* = −1.3 kcal/mol), while the presence of more electron-donating groups (entries 3 and 4) leads to slightly higher δ*E* due to the destabilization of **TS**_**Isom_3**_ (ΔΔ*G*^‡^ = +0.8 kcal/mol) such as in entry 3 or a stabilization of **Λ-*cis*A** (ΔΔ*G* = −1.3 kcal/mol) such as in entry 4. Furthermore, a CF_3_ group in para to the oxygen (entry 5) destabilizes **TS_Isom_3_**, resulting in a larger δ*E* (37.2 vs 34.4 kcal/mol without substituents), whereas an electron-donating group such as Me (entry 6) does not influence much the reactivity (Δδ*E* = +0.3 kcal/mol). On the other hand, the presence of an electron-donating phosphine (entries 7 and 8) predicts better catalysts due to a lower δ*E* (31.1 and 32.8 kcal/mol for **7** and **8** vs 34.4 kcal/mol for **1**), resulting from the stabilization of species **TS**_**Isom_3**_. Experimentally, Jia and co-workers analyzed the kinetics of the homometathesis using 1-methoxy-4-(1-propyn-1-yl)benzene and catalysts **1–3**, **5**, and **7**.^31^ Similar kinetics were obtained for catalysts **1**–**3** and **5**, while **7** was the best catalyst. Although we modeled the reaction using acetylene as the reactant, our computational results show similar δ*E*s for catalysts **1**–**3** and **5** (34.4, 34.0, 35.2, and 37.2 kcal/mol, respectively). At the same time, the δ*E* for **7** is lowered to 31.1 kcal/mol. With this, our results nicely fit with the experimental evidence, as catalysts present similar kinetics, except for the catalyst bearing cyclohexylphosphine, which in both studies presented faster catalysis.

**Table 1:** Ligand Substitution Scope (See Scheme 2b) for the Alkyne Metathesis Reaction Studied^a^

entry	R_1_	R_2_	Λ-*cis* A	TS_Isom_3_	δ*E*
1^a^	H	Ph	–4.9	29.5	34.4
2^a^	H	(*p*-CF_3_)Ph	–6.2	27.8	34.0
3^a^	H	(*p*-OMe)Ph	–4.9	30.3	35.2
4	H	(*p*-Me)Ph	–6.5	29.0	35.5
5^a^	CF_3_	Ph	–4.6	32.6	37.2
6	Me	Ph	–4.5	30.2	34.7
7^a^	H	Cy	–4.4	26.7	31.1
8	H	Me	–5.4	27.4	32.8

a Gibbs energies in kcal/mol. a = catalyst tested experimentally by Jia et al.

Overall, no clear trends are extracted solely on the basis of modifying the electronic character (withdrawing vs donating) of the substituents. Instead, and as already pinpointed in the literature,^48^ the tris-chelate metal complexes isomerization is influenced by the bite angle of the ligand. First, the stability of **Λ-*cis*A** has been correlated with one O–Re–P bite angle of **Λ-*cis*A** (*R*^2^ = 0.890). Simultaneously, δ*E* presents a rather large linear correlation with the same bite angle (*R*^2^ = 0.759), the one with the alkyne–Re–C_alkylidyne_ angle in between (*R*^2^ = 0.842, R_2_ = Cy excluded from correlation). Considering the two variables together, the correlation improves (*R*^2^ = 0.784). The graphical representation of the linear regressions is provided in Figures S6–S8. Again, the influence of this angle can be rationalized by visual inspection of the molecular orbitals of **Λ-*cis*A**. The HOMO is delocalized over the O–Re–P bite angle; meanwhile, the alkyne–Re–C_alkylidyne_ is distorted due to the repulsion between the π systems electron cloud of alkyne and alkylidyne.

**Figure 5:**
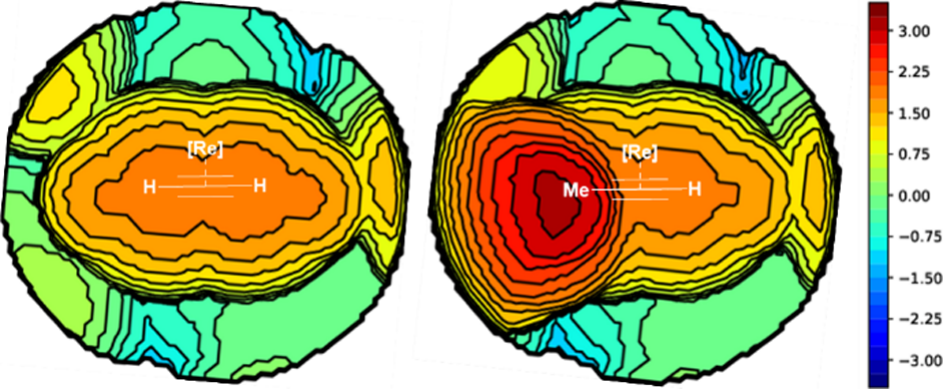
Steric maps of species **Λ-*cis*A** for the alkynes: (a) acetylene and (b) propyne, with the corresponding %*V*_Bur_ values on the *xy* plane (using the two carbon atoms of the alkyne as center, with a radius of 3.5 Å and with the *z*-axis defined by the metal; the *xy* plane is perpendicular to the *z*-axis and contains both carbon atoms of the alkyne, including the H atoms). Isocontour curves are given in Å.

Finally, the effect of substituents on the alkyne has been evaluated in a predictive way.^33,49^ In the case of using propyne instead of acetylene, **TS**_**Isom_3**_ is destabilized (31.4 vs 29.5 kcal/mol with acetylene), and also **Λ-*cis*A** results in destabilization (1.7 vs −4.9 kcal/mol) due to the larger steric hindrance. The P–Re–P angle is distorted increasing the steric hindrance of the substituents of the simple acetylene, going from 157.3 to 155.2° for propyne. Going to an analysis of the steric hindrance using the steric maps of Cavallo and co-workers,^50^ it was found that at the midpoint of the two carbons of the alkyne entering the intermediate **Λ-*cis*A**, the %*V*_Bur_ increases from 65.3 to 71.5% (Figure 5). This is logical, but the difference should have been smaller due to the distortion of the P–Re–P angle to accommodate the alkyne mentioned above. However, a full understanding of the substituent effect is required for further studies.

## Conclusions

In summary, our study presents a comprehensive mechanistic investigation of Re(V)-based alkyne metathesis, building upon the experimental findings reported by Jia and co-workers.^31^ DFT calculations revealed that the reaction initiates with the dissociation of the pyridine, followed by the entry of an alkyne molecule via a stepwise mechanism. However, the reaction necessitates an isomerization process, as the activation pathway in the retrocycloaddition step is kinetically impossible under the experimental conditions (44.5 kcal/mol). Interestingly, the reaction can effectively proceed through the **Λ-*cis*A** stereoisomer without a prohibitive overall activation energy barrier.

Further exploration of all possible isomerization pathways revealed that while the isomerization becomes hindered when the pyridine is bonded, the process is significantly facilitated upon its exchange with an alkyne. With this, **TS**_**Isom_3**_ results being the reaction TDTS. Experimental evidence suggests that the TDTS is the pyridine dissociation, and although most of the DFT calculations are performed with the simplified acetylene, our results corroborate well with the reported experimental kinetics. In fact, aryl phosphines provide similar kinetics, and the cyclohexylphosphine catalyst is the best catalyst. It is worth mentioning that the faster the pyridine leaves, the faster the catalyst can isomerize.

Moreover, our results revealed that the isomerization seems to depend on the phosphine-phenolate bite angles in **Λ-*cis*A**, whereas adding substituents on acetylene seems destabilizing slightly **TS**_**Isom_3**_ and much more **Λ-*cis*A** due to the higher steric hindrance between the methyl substituent and the phenyl phosphine.

## Supporting Information Available

Coordinates for all reactants, intermediates, and transition states (XYZ)
Frontier molecular orbital analysis of selected species, 3D-view of selected transition states, relative energies and selected angles for the crucial species of the ligand substitution scope, and plots of the linear correlations attempted in the study (PDF)

## Supplementary Material

ic3c04235_si_001.xyz

## References

[ref1] Haibach M. C.; Kundu S.; Brookhart M.; Goldman A. S. Alkane Metathesis by Tandem Alkane-Dehydrogenation–Olefin-Metathesis Catalysis and Related Chemistry. Acc. Chem. Res. 2012, 45, 947–958. 10.1021/ar3000713. 22584036

[ref2] Bailey B. C.; Schrock R. R.; Kundu S.; Goldman A. S.; Huang Z.; Brookhart M. Evaluation of molybdenum and tungsten metathesis catalysts for homogeneous tandem alkane metathesis. Organometallics 2009, 28, 355–360. 10.1021/om800877q.

[ref3] aHérisson P. J. L.; Chauvin Y. Catalyse de transformation des oléfines par les complexes du tungstène. II. Télomérisation des oléfines cycliques en présence d’oléfines acycliques. Die Makromol. Chem. 1971, 141, 161–176. 10.1002/macp.1971.021410112.

[ref4] aSchrock R. R. Multiple Metal-Carbon Bonds for Catalytic Metathesis Reactions (Nobel Lecture). Angew. Chem., Int. Ed. 2006, 45, 3748–3759. 10.1002/anie.200600085. 16703641

[ref5] aGrubbs R. H.; Wenzel A. G.; O’Leary D. J.; Khosravi E. Handbook of Metathesis; John Wiley & Sons, 2015.

[ref6] aFürstner A. The Ascent of Alkyne Metathesis to Strategy-Level Status. J. Am. Chem. Soc. 2021, 143, 15538–15555. 10.1021/jacs.1c08040. 34519486 PMC8485352

[ref7] Ehrhorn H.; Tamm M. Well-Defined Alkyne Metathesis Catalysts: Developments and Recent Applications. Chem.—Eur. J. 2019, 25, 3190–3208. 10.1002/chem.201804511. 30346054

[ref8] aYiannakas E.; Grimes M. I.; Whitelegge J. T.; Fürstner A.; Hulme A. N. An Alkyne-Metathesis-Based Approach to the Synthesis of the Anti-Malarial Macrodiolide Samroiyotmycin A. Angew. Chem., Int. Ed. 2021, 60, 18504–18508. 10.1002/anie.202105732. PMC8456858 34076945

[ref9] aKiel G. R.; Bay K. L.; Samkian A. E.; Schuster N. J.; Lin J. B.; Handford R. C.; Nuckolls C.; Houk K. N.; Tilley T. D. Expanded Helicenes as Synthons for Chiral Macrocyclic Nanocarbons. J. Am. Chem. Soc. 2020, 142, 11084–11091. 10.1021/jacs.0c03177. 32450694

[ref10] avon Kugelgen S.; Piskun I.; Griffin J. H.; Eckdahl C. T.; Jarenwattananon N. N.; Fischer F. R. Templated Synthesis of EndFunctionalized Graphene Nanoribbons through Living Ring-Opening Alkyne Metathesis Polymerization. J. Am. Chem. Soc. 2019, 141, 11050–11058. 10.1021/jacs.9b01805. 31264864

[ref11] aFürstner A. Lessons from Natural Product Total Synthesis: Macrocyclization and Postcyclization Strategies. Acc. Chem. Res. 2021, 54, 861–874. 10.1021/acs.accounts.0c00759. 33507727 PMC7893715

[ref12] aZhang W.; Moore J. S. Synthesis of Poly(2,5-thienyleneethynylene)s by Alkyne Metathesis. Macromolecules 2004, 37, 3973–3975. 10.1021/ma049371g.

[ref13] aWengrovius J. H.; Sancho J.; Schrock R. R. Metathesis of acetylenes by tungsten(VI)-alkylidyne complexes. J. Am. Chem. Soc. 1981, 103, 3932–3934. 10.1021/ja00403a058.

[ref14] Pennella F.; Banks R. L.; Bailey G. C. Disproportionation of alkynes. Chem. Commun. 1968, 1548–1549. 10.1039/c19680001548.

[ref15] aMortreux A.; Blanchard M. Metathesis of alkynes by a molybdenum hexacarbonyl-resorcinol catalyst. J. Chem. Soc., Chem. Commun. 1974, 786–787. 10.1039/C39740000786.

[ref16] aHaberlag B.; Freytag M.; Daniliuc C. G.; Jones P. G.; Tamm M. Efficient metathesis of terminal alkynes. Angew. Chem., Int. Ed. 2012, 51, 13019–13022. 10.1002/anie.201207772. 23161650

[ref17] aBindl M.; Stade R.; Heilmann E. K.; Picot A.; Goddard R.; Fürstner A. Molybdenum nitride complexes with Ph_3_SiO ligands are exceedingly practical and tolerant precatalysts for alkyne metathesis and efficient nitrogen transfer agents. J. Am. Chem. Soc. 2009, 131, 9468–9470. 10.1021/ja903259g. 19534524

[ref18] aBeer S.; Hrib C. G.; Jones P. G.; Brandhorst K.; Grunenberg J.; Tamm M. Efficient room-temperature alkyne metathesis with well-defined imidazolin-2-iminato tungsten alkylidyne complexes. Angew. Chem., Int. Ed. 2007, 46, 8890–8894. 10.1002/anie.200703184. 17935104

[ref19] Melcher D.; Àrias Ò.; Freytag M.; Jones P. G.; Tamm M. Synthesis of Alkyne Metathesis Catalysts from Tris(dimethylamido) tungsten Precursors. Eur. J. Inorg. Chem. 2020, 2020, 4454–4464. 10.1002/ejic.202000835.

[ref20] aEstes D. P.; Gordon C. P.; Fedorov A.; Liao W.-C.; Ehrhorn H.; Bittner C.; Zier M. L.; Bockfeld D.; Chan K. W.; Eisenstein O.; Raynaud C.; Tamm M.; Copéret C. Molecular and Silica-Supported Molybdenum Alkyne Metathesis Catalysts: Influence of Electronics and Dynamics on Activity Revealed by Kinetics, SolidState NMR, and Chemical Shift Analysis. J. Am. Chem. Soc. 2017, 139, 17597–17607. 10.1021/jacs.7b09934. 29083916

[ref21] aGroos J.; Hauser P. M.; Koy M.; Frey W.; Buchmeiser M. R. Highly Reactive Cationic Molybdenum Alkylidyne N-Heterocyclic Carbene Catalysts for Alkyne Metathesis. Organometallics 2021, 40, 1178–1184. 10.1021/acs.organomet.1c00175.

[ref22] aZhang W.; Kraft S.; Moore J. S. Highly active trialkoxymolybdenum(VI) alkylidyne catalysts synthesized by a reductive recycle strategy. J. Am. Chem. Soc. 2004, 126, 329–335. 10.1021/ja0379868. 14709099

[ref23] aJyothish K.; Zhang W. Introducing a podand motif to alkyne metathesis catalyst design: a highly active multidentate molybdenum(VI) catalyst that resists alkyne polymerization. Angew. Chem., Int. Ed. 2011, 50, 3435–3438. 10.1002/anie.201007559. 21394862

[ref24] aHillenbrand J.; Leutzsch M.; Fürstner A. Molybdenum Alkylidyne Complexes with Tripodal Silanolate Ligands: The Next Generation of Alkyne Metathesis Catalysts. Angew. Chem., Int. Ed. 2019, 58, 15690–15696. 10.1002/anie.201908571. PMC6856820 31449713

[ref25] aThompson R. R.; Rotella M. E.; Du P.; Zhou X.; Fronczek F. R.; Kumar R.; Gutierrez O.; Lee S. Siloxide Podand Ligand as a Scaffold for Molybdenum-Catalyzed Alkyne Metathesis and Isolation of a Dynamic Metallatetrahedrane Intermediate. Organometallics 2019, 38, 4054–4059. 10.1021/acs.organomet.9b00430.

[ref26] Ge Y.; Huang S.; Hu Y.; Zhang L.; He L.; Krajewski S.; Ortiz M.; Jin Y.; Zhang W. Highly active alkyne metathesis catalysts operating under open air condition. Nat. Commun. 2021, 12, 1136 10.1038/s41467-021-21364-4. 33602910 PMC7893043

[ref27] Schrock R. R. High Oxidation State Molybdenum and Tungsten Alkene and Alkyne Metathesis Catalysts: Where We Are and Where We Want to Go. Adv. Synth. Catal. 2007, 349, 25–25. 10.1002/adsc.200600619.

[ref28] Mayr A.; Hoffmeister H. Recent advances in the chemistry of metal-carbon triple bonds. Adv. Organomet. Chem. 1991, 32, 227–324. 10.1016/S0065-3055(08)60481-5.

[ref29] aChen S.; Liu L.; Gao X.; Hua Y.; Peng L.; Zhang Y.; Yang L.; Tan Y.; He F.; Xia H. Addition of alkynes and osmium carbynes towards functionalized dπ-pπ conjugated systems. Nat. Commun. 2020, 11, 4651 10.1038/s41467-020-18498-2. 32938934 PMC7495419

[ref30] Cui M.; Bai W.; Sung H. H. Y.; Williams I. D.; Jia G. Robust Alkyne Metathesis Catalyzed by Air Stable d^2^ Re(V) Alkylidyne Complexes. J. Am. Chem. Soc. 2020, 142, 13339–13344. 10.1021/jacs.0c06581. 32673485

[ref31] Cui M.; Sung H. H. Y.; Williams I. D.; Jia G. Alkyne Metathesis with d^2^ Re(V) Alkylidyne Complexes Supported by Phosphino-Phenolates: Ligand Effect on Catalytic Activity and Applications in Ring-Closing Alkyne Metathesis. J. Am. Chem. Soc. 2022, 144, 6349–6360. 10.1021/jacs.2c00368. 35377156

[ref32] Cui M.; Jia G. Organometallic Chemistry of Transition Metal Alkylidyne Complexes Centered at Metathesis Reactions. J. Am. Chem. Soc. 2022, 144, 12546–12566. 10.1021/jacs.2c01192. 35793547

[ref33] Monreal-Corona R.; Pla-Quintana A.; Poater A. Predictive catalysis: a valuable step towards machine learning. Trends Chem. 2023, 5, 935 10.1016/j.trechm.2023.10.005.

[ref34] Frisch M. J.; Trucks G. W.; Schlegel H. B.; Scuseria G. E.; Robb M. A.; Cheeseman J. R.; Scalmani G.; Barone V.; Mennucci B.; Petersson G. A.; Nakatsuji H.; Caricato M.; Li X.; Hratchian H. P.; Izmaylov A. F.; Bloino J.; Zheng G.; Sonnenberg J. L.; Hada M.; Ehara M.; Toyota K.; Fukuda R.; Hasegawa J.; Ishida M.; Nakajima T.; Honda Y.; Kitao O.; Nakai H.; Vreven T.; Montgomery J. A. Jr.; Peralta J. E.; Ogliaro F.; Bearpark M.; Heyd J. J.; Brothers E.; Kudin K. N.; Staroverov V. N.; Kobayashi R.; Normand J.; Raghavachari K.; Rendell A.; Burant J. C.; Iyengar S. S.; Tomasi J.; Cossi M.; Rega N.; Millam J. M.; Klene M.; Knox J. E.; Cross J. B.; Bakken V.; Adamo C.; Jaramillo J.; Gomperts R.; Stratmann R. E.; Yazyev O.; Austin A. J.; Cammi R.; Pomelli C.; Ochterski J. W.; Martin R. L.; Morokuma K.; Zakrzewski V. G.; Voth G. A.; Salvador P.; Dannenberg J. J.; Dapprich S.; Daniels A. D.; Farkas Ö.; Foresman J. B.; Ortiz J. V.; Cioslowski J.; Fox D. J. Gaussian 09, Revision E.01; Gaussian, Inc.: Wallingford, CT, 2009.

[ref35] aBecke A. Density-functional Exchange-Energy Approximation with Correct Asymptotic Behavior. Phys. Rev. A: At., Mol., Opt. Phys. 1988, 38, 3098–3100. 10.1103/PhysRevA.38.3098. 9900728

[ref36] Schäfer A.; Huber C.; Ahlrichs R. Fully optimized contracted Gaussian basis sets of triple zeta valence quality for atoms Li to Kr. J. Chem. Phys. 1994, 100, 5829 10.1063/1.467146.

[ref37] aKüchle W.; Dolg M.; Stoll H.; Preuss H. Energy-adjusted pseudopotentials for the actinides. Parameter sets and test calculations for thorium and thorium monoxide. J. Chem. Phys. 1994, 100, 7535–7542. 10.1063/1.466847.

[ref38] Zhao Y.; Truhlar D. G. The M06 suite of density functionals for main group thermochemistry, thermochemical kinetics, noncovalent interactions, excited states, and transition elements: two new functionals and systematic testing of four M06-class functionals and 12 other functionals. Theor. Chem. Acc. 2008, 120, 215–241. 10.1007/s00214-007-0310-x.

[ref39] Weigend F.; Ahlrichs R. Balanced basis sets of split valence, triple zeta valence and quadruple zeta valence quality for H to Rn: Design and assessment of accuracy. Phys. Chem. Chem. Phys. 2005, 7, 3297–3305. 10.1039/b508541a. 16240044

[ref40] Marenich A. V.; Cramer C. J.; Truhlar D. G. Universal Solvation Model Based on Solute Electron Density and on a Continuum Model of the Solvent Defined by the Bulk Dielectric Constant and Atomic Surface Tensions. J. Phys. Chem. B 2009, 113, 6378–6396. 10.1021/jp810292n. 19366259

[ref41] aAtagi L. M.; Critchlow S. C.; Mayer J. M. Reactivity of the tungsten carbyne W(≡CCH_3_)Cl(PMe_3_)_4_: Double carbonylation, carbyne-alkyne complexes, and stoichiometric acetylene metathesis. J. Am. Chem. Soc. 1992, 114, 9223–9224. 10.1021/ja00049a085.

[ref42] aZhu C.; Yang Y.; Luo M.; Yang C.; Wu J.; Chen L.; Liu G.; Wen T.; Zhu J.; Xia H. Stabilizing Two Classical Antiaromatic Frameworks: Demonstration of Photoacoustic Imaging and the Photothermal Effect in Metallaaromatics. Angew. Chem., Int. Ed. 2015, 54, 6181–6185. 10.1002/anie.201501349. 25824395

[ref43] aMayer I. Charge, bond order and valence in the AB initio SCF theory. Chem. Phys. Lett. 1983, 97, 270–274. 10.1016/0009-2614(83)80005-0.

[ref44] aPoater J.; Gimferrer M.; Poater A. Covalent and Ionic Capacity of MOFs To Sorb Small Gas Molecules. Inorg. Chem. 2018, 57, 6981–6990. 10.1021/acs.inorgchem.8b00670. 29799198

[ref45] aAhmadi M.; Panahi F.; Bahri-Laleh N.; Sabzi M.; Pareras G.; Falcone B. N.; Poater A. pH-Responsive Gelation in Metallo-Supramolecular Polymers Based on the Protic Pyridinedicarboxamide Ligand. Chem. Mater. 2022, 13, 6155–6169. 10.1021/acs.chemmater.2c01346.

[ref46] aRây P.; Dutt N. K. Kinetics and mechanism of racemisation of optically active cobaltic tris biguanide complex. J. Indian Chem. Soc. 1943, 20, 8192 10.5281/zenodo.6598283.

[ref47] Kozuch S.; Shaik S. How to Conceptualize Catalytic Cycles? The Energetic Span Model. Acc. Chem. Res. 2011, 44, 101–110. 10.1021/ar1000956. 21067215

[ref48] aDavis A. V.; Firman T. K.; Hay B. P.; Raymond K. N. d-Orbital Effects on Stereochemical Non-Rigidity: Twisted Ti^IV^ Intramolecular Dynamics. J. Am. Chem. Soc. 2006, 128, 9484–9496. 10.1021/ja0617946. 16848486

[ref49] aMaloney M. P.; Stenfors B. A.; Helquist P.; Norrby P.-O.; Wiest O. Interplay of Computation and Experiment in Enantioselective Catalysis: Rationalization, Prediction, and—Correction?. ACS Catal. 2023, 13, 14285–14299. 10.1021/acscatal.3c03921.

[ref50] aPoater A.; Cosenza B.; Correa A.; Giudice S.; Ragone F.; Scarano V.; Cavallo L. SambVca: A Web Application for the Calculation of the Buried Volume of N-Heterocyclic Carbene Ligands. Eur. J. Inorg. Chem. 2009, 2009, 1759–1766. 10.1002/ejic.200801160.

