# Supplementary material for: Rhenium Alkyne Catalysis: Sterics Control the Reactivity
Source: Inorg Chem. 2024 Mar 20;63(13):5842–51. doi: 10.1021/acs.inorgchem.3c04235 (PMC10988556; doi:10.1021/acs.inorgchem.3c04235)
Supplement: Supplementary file 2 — ic3c04235_si_002.pdf [file ic3c04235_si_002.pdf]

Supporting Information for: “Rhenium Alkyne Catalysis: Sterics Control the Reactivity”

Michele Tomasini,[a,b] *Martí Gimferrer,*[c,*] *Lucia Caporaso,*[b,d,*] *and Albert Poater*[a,*]

a) Institut de Química Computacional i Catàlisi i Departament de Química, Universitat de Girona, c/ Maria Aurèlia Capmany i Farnés 69, 17003, Girona, Catalonia, Spain.

b) Dipartimento di Chimica e Biologia, Università di Salerno, Via Ponte don Melillo, 84084, Fisciano, Italy.

c) Institut für Physikalische Chemie, Georg-August Universität Göttingen, Tammannstraße 6, 37077 Göttingen, Germany.

d) CIRCC, Interuniversity Consortium Chemical Reactivity and Catalysis, via Celso Ulpiani 27, 70126 Bari, Italy.

Email: marti.gimferrerandres@uni-goettingen.de, lcaporaso@unisa.it, albert.poater@udg.edu

TABLE OF CONTENTS

Figures S1-S2. Frontier molecular orbital analysis of species **Δ-cis A** and **Λ-cis A**.

Figures S3-S5. 3D-view of species $\mathbf{TS_{Isom\_1}}$, $\mathbf{TS_{Isom\_2}}$ and $\mathbf{TS_{Isom\_3}}$.

Table S1. Relative energies and selected angles for the crucial species of the ligand substitution scope.

Figures S6-S8. Plots of the linear correlations attempted in the study.

Δ-cis A

HOMO – 1

Top view

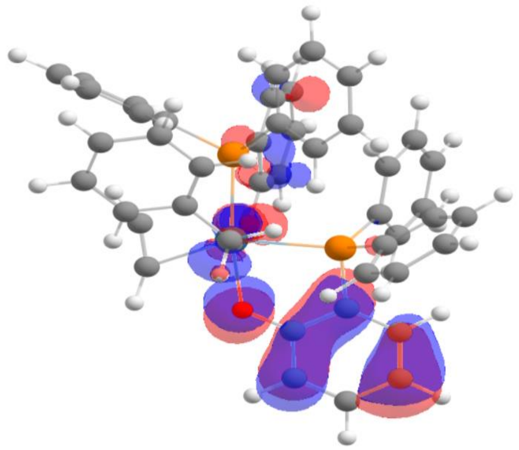

Side view

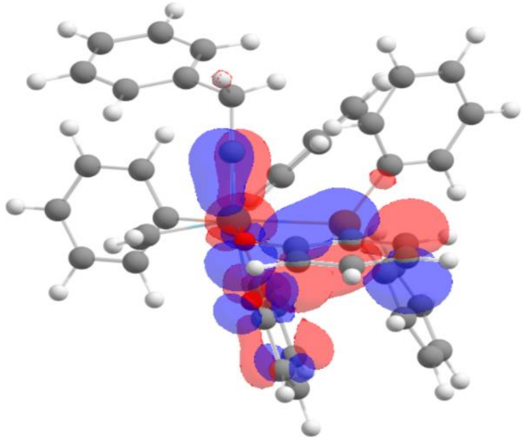

HOMO

Top view

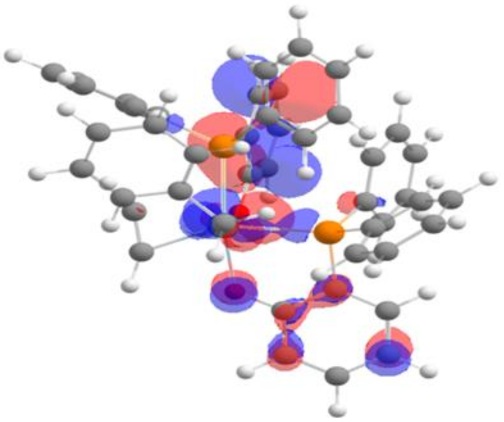

Side view

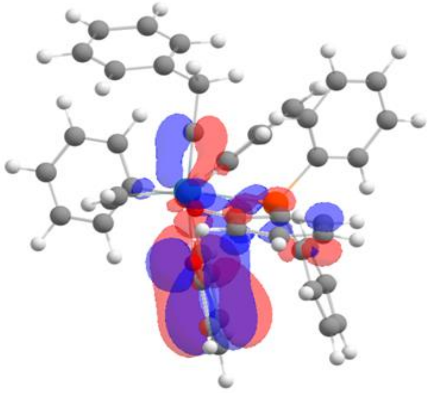

LUMO

Top view

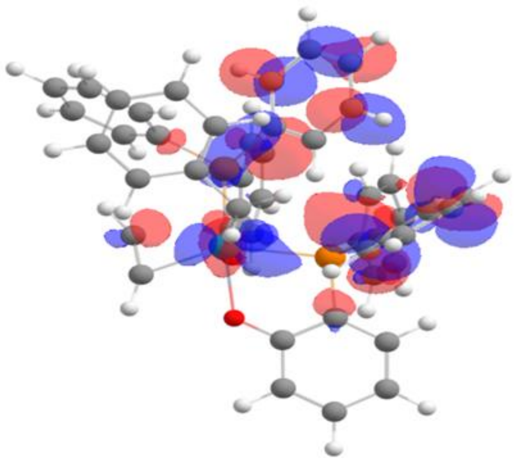

Side view

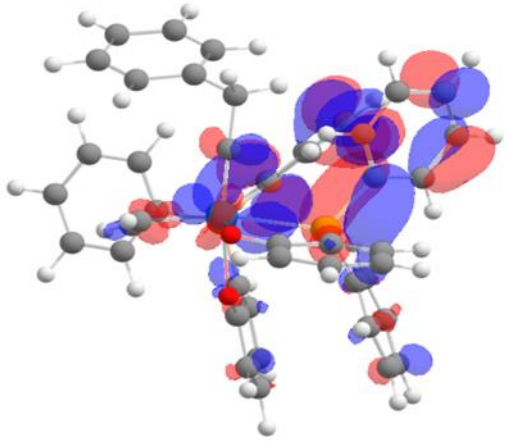

Figure S1. Frontier molecular orbitals for intermediate **Δ-cis A**. Isocontour value selected for the orbital representation of 0.03.

Λ-cis A

HOMO - 1

Top view

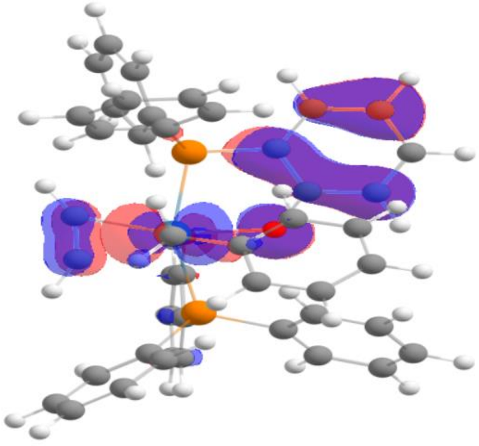

Side view

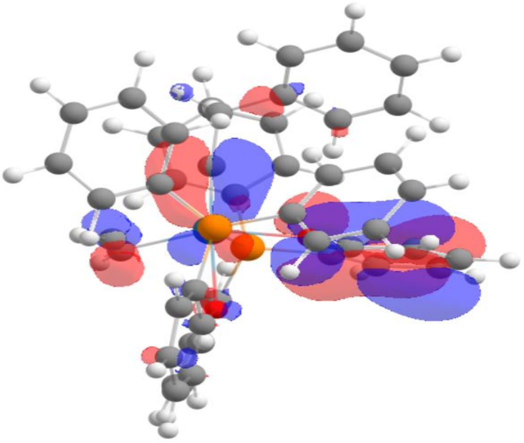

HOMO

Top view

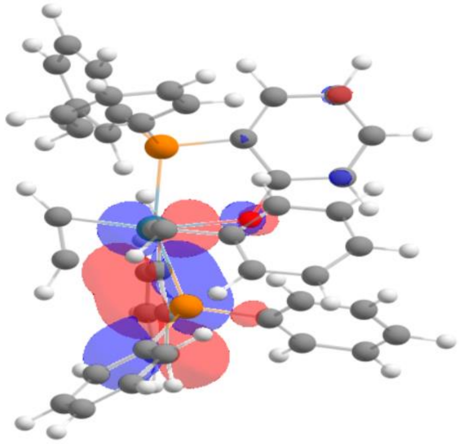

Side view

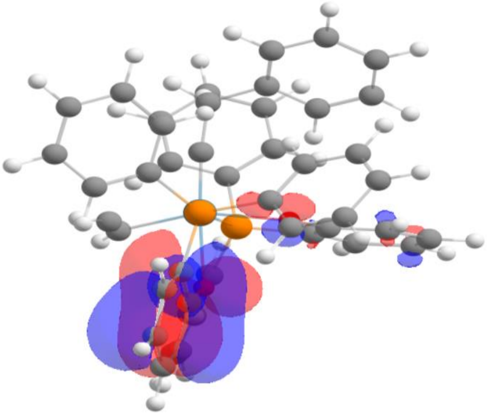

LUMO

Top view

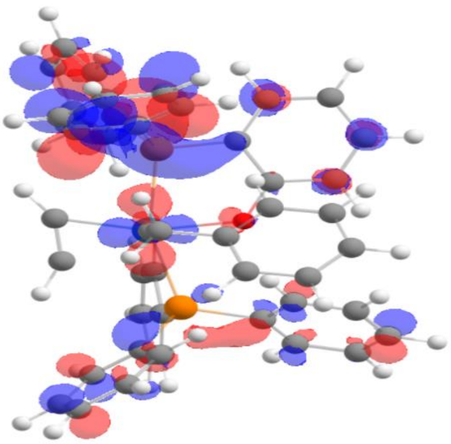

Side view

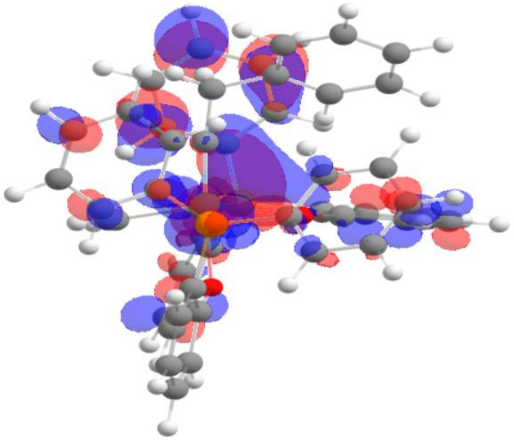

Figure S2. Frontier molecular orbitals for intermediate **Λ-cis A**. Isocontour value selected for the orbital representation of 0.03.

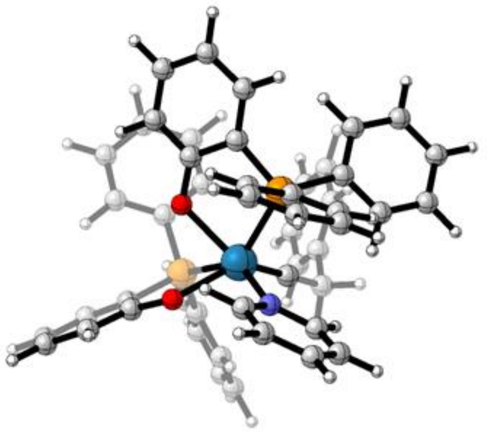

Figure S3. 3D-view of the transition state $\mathbf{TS_{Isom\_1}}$ for isomerization of **R**.

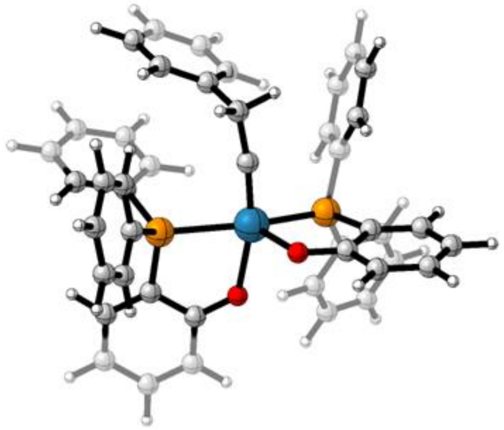

Figure S4. 3D-view of the transition state $\mathbf{TS_{Isom\_2}}$ for isomerization of **A0**.

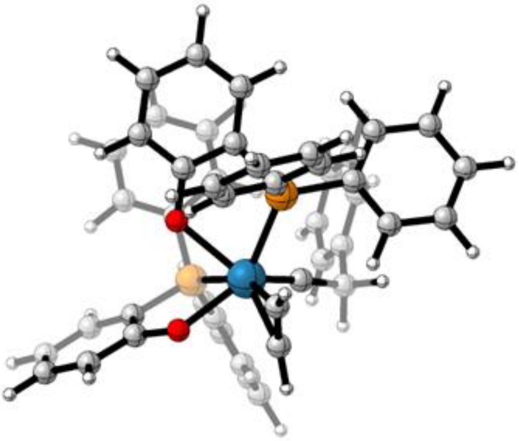

Figure S5. 3D-view of the transition state $\mathbf{TS_{Isom\_3}}$ for isomerization of **Δ-cis A**.

Table S1. Ligand substitution scope (see Table 1 in the main manuscript) and selected angles. Relative energies and angles are reported in kcal/mol and degrees, respectively.

Entry	Λ-cis A	TS_{Isom_3}	δE	Alkyne-Re-$C_{alkylidine}$ angle	O-Re-P angle
1	-4.9	29.5	34.4	100.6	78.7
2	-6.2	27.8	34.0	100.4	78.4
3	-4.9	30.3	35.2	100.6	78.7
4	-6.5	29.0	35.5	100.6	78.7
5	-4.6	32.6	37.2	100.8	78.8
6	-4.5	30.2	34.7	100.6	78.7
7	-13.2	18.0	31.1	101.1	77.3
8	-5.4	27.4	32.8	102.1	78.3

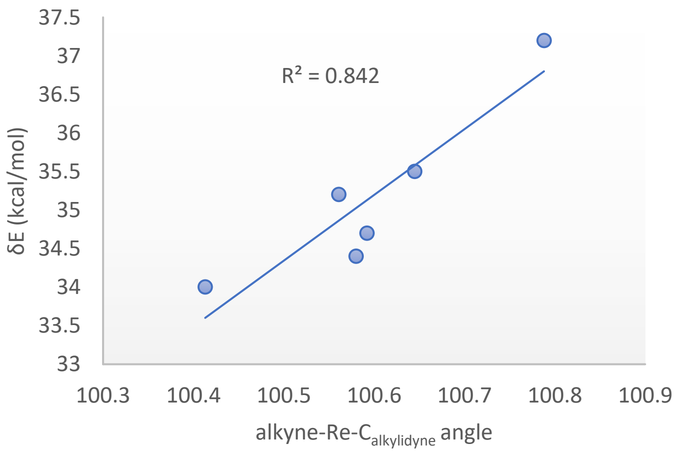

Figure S6. Plot of δE vs alkyne-Re-$C_{alkylidyne}$ angle (entries 7 and 8 not considered). The alkyne-Re- $C_{alkylidyne}$ angle is determined by the following atoms: the midpoint between the alkyne carbon atoms, Re and $C_{alkylidyne}$.

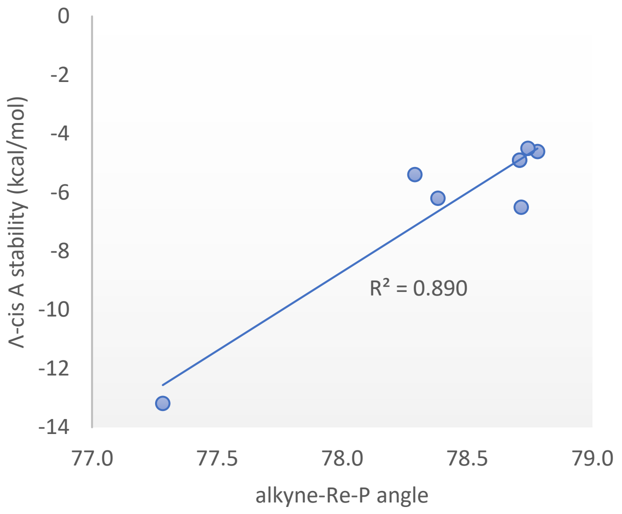

Figure S7. Plot of **Λ-cis A** energy vs alkyne-Re-$C_{alkylidyne}$ angle.

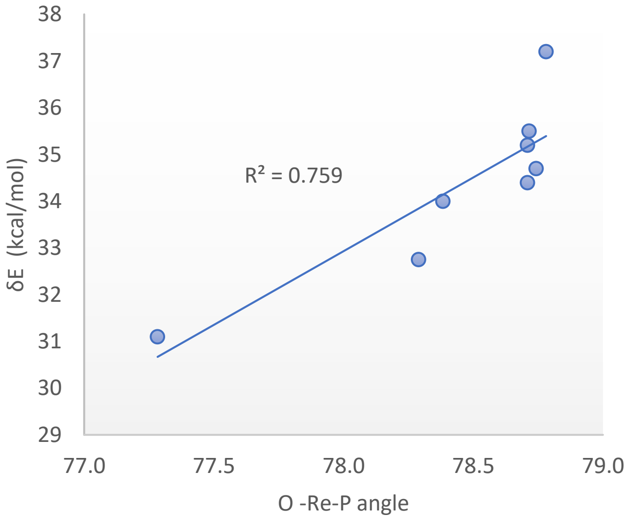

Figure S8. Plot of δE vs O-Re-P angle.
